# Increased Stretching of Mechanoreceptors in Superior Tarsal Muscle Reflexively Contracts Upper Trapezius Muscle as well as Levator Palpebrae Superioris and Occipitofrontalis Muscles as Eye-Eyelid-Eyebrow-Head Coordinated Movements: A Case Series

**DOI:** 10.7759/cureus.80743

**Published:** 2025-03-17

**Authors:** Kiyoshi Matsuo, Ai Kaneko, Tae Otsuka

**Affiliations:** 1 Plastic Surgery/Oculoplastic Surgery, Matsuo Plastic and Oculoplastic Surgery Clinic, Hamamatsu, JPN; 2 Plastic and Reconstructive Surgery, Shinshu University School of Medicine, Matsumoto, JPN; 3 Psychiatry, Apricot Co. Ltd., Yokohama, JPN

**Keywords:** eye-eyelid-eyebrow-head coordinated movements, forward head posture, head extending backward, levator palpebrae superioris muscle, neck pain, occipitofrontalis muscle, rostral locus coeruleus, superior tarsal muscle, tension-type headache, upper trapezius muscle

## Abstract

To maintain a vertical visual field, fast-twitch fibers in the levator palpebrae superioris muscle (LPSM) stretch mechanoreceptors in the superior tarsal muscle (STM), which contracts the slow-twitch fibers in both the LPSM and the occipitofrontalis muscle (OFM). Exceeding the upgaze limit without head movement increases reflex contraction of the OFM to raise the eyebrows and pull the scalp backward while also causing involuntary contraction of the upper trapezius muscle (UTM) to extend the head backward, resulting in tension-type headaches (TTH) and neck pain. Due to aponeurosis disinsertion from the tarsus, we hypothesized that increased mechanoreceptor stretching in the STM reflexively contracts both the OFM and UTM. We report a case series of five patients whose aponeurosis disinsertion caused tonic eyebrow-raising, TTH, and neck pain. In the first case, asymmetrical disinsertion with dominance on the left side led to a more pronounced contraction of the OFM and UTM, resulting in TTH and neck pain on that side. After surgery to reduce mechanoreceptor stretching, symptoms resolved. The second and third cases, which involved symmetrical disinsertion, showed that unilateral eyebrow lifting using tape to alleviate mechanoreceptor stretching reduced ipsilateral UTM contraction in length and hardness. The fourth case, also with symmetrical disinsertion, experienced symptom relief post-surgery. In the fifth case, the eyebrows were maximally raised due to severe aponeurosis disinsertion, and the head was extended backward and protruded. Bilateral aponeurosis reinsertion lowered the eyebrows and reduced the extension/protrusion of the head, relieving TTH and neck pain. These cases suggest that increased mechanoreceptor stretching in the STM reflexively contracts the slow-twitch fibers of the OFM and UTM, contributing to TTH and neck pain as part of coordinated eye, eyebrow, and head movements.

## Introduction

To maintain a vertical visual field without moving the head, voluntary contractions and microsaccades of fast-twitch fibers in the levator palpebrae superioris muscle (LPSM) and the global layer of the superior rectus muscle (GLSRM) [1, 2] stretch mechanoreceptors in the superior tarsal muscle (STM) [3, 4] in a degree-dependent manner. This stretching leads to the contraction of slow-twitch fibers [5] in the LPSM [6] and occipitofrontalis muscle [7-9], resulting in coordinated movements of the eyes, eyelids, and eyebrows via the mesencephalic trigeminal nucleus (MTN), rostral locus coeruleus [10], and brainstem motor centers [11].

To maintain primary gaze, voluntary contractions and microsaccades of fast-twitch fibers in the LPSM and GLSRM [1, 2] stretch the mechanoreceptors in the STM [3, 4]. This induces phasic reflex contractions of the slow-twitch fibers [5] in the LPSM [6] and frontalis muscle [7, 8], facilitating the retraction of the upper eyelid and the elevation of the eyebrow as part of coordinated eye-eyelid-eyebrow movements via the MTN and brainstem motor centers.

To maintain a 30° upgaze without moving the head, increased voluntary contractions and microsaccades of the fast-twitch fibers in the LPSM and GLSRM [1, 2] further stretch the mechanoreceptors in the STM [3, 4, 12], enhancing phasic reflex contractions of the slow-twitch fibers [5] in the LPSM [6] and frontalis muscle [7, 8]. This results in a more significant retraction of the upper eyelid and elevation of the eyebrow, continuing the coordinated movements via the MTN and brainstem motor centers.

To maintain a 60° upgaze without moving the head, even greater voluntary contractions and microsaccades of the fast-twitch fibers in the LPSM and GLSRM [1, 2] significantly stretch the mechanoreceptors in the STM [3, 4, 12], leading to an increase in phasic reflex contractions of the slow-twitch fibers [5] in the LPSM [6] and frontalis muscle [7-9]. This stretching also induces tonic reflex contractions of slow-twitch fibers in the occipitofrontalis [7-9], orbital orbicularis oculi [13, 14], and facial expression muscles [12], which raise the eyebrow tonically, pull the scalp backward, and create glabellar grimacing. This results in coordinated movements of the eye, eyelid, eyebrow, scalp, and face [7-9, 12-14] via the MTN, rostral locus coeruleus [10], and brainstem motor centers [11]. However, most people cannot maintain a 60° upgaze. When the limit of upgaze is exceeded without moving the head, the head is spontaneously extended backward due to involuntary contraction of the upper trapezius muscle.

Habitual rubbing and stretching of the upper eyelid, such as when applying contact lenses or eyedrops, can easily disinsert the levator aponeurosis from the tarsus [12, 15, 16]. In patients with aponeurosis disinsertion, primary gaze leads to increased mechanoreceptor stretching [3, 4], which corresponds to upgaze in normal individuals, inducing tonic reflex contraction of the occipitofrontalis muscle [7-9] to raise the eyebrow, often resulting in tension-type headaches (TTH) [9]. Patients with TTH frequently complain of neck pain caused by increased involuntary contraction of slow-twitch fibers in the upper trapezius muscle [17].

Thus, we hypothesized that increased stretching of mechanoreceptors in the STM reflexively and tonically contracts slow-twitch fibers in the upper trapezius muscle, contributing to neck pain and affecting the occipitofrontalis muscle, which leads to TTH. To verify this hypothesis, we report a case series of five patients with increased mechanoreceptor stretching due to aponeurosis disinsertion, eyebrow elevation with TTH, and neck pain.

This report was previously presented as two oral speeches at the 62nd Annual Meeting of the Japan Society of Plastic and Reconstructive Surgery on May 15-17th, 2019.

## Case presentation

**Figure 1:**
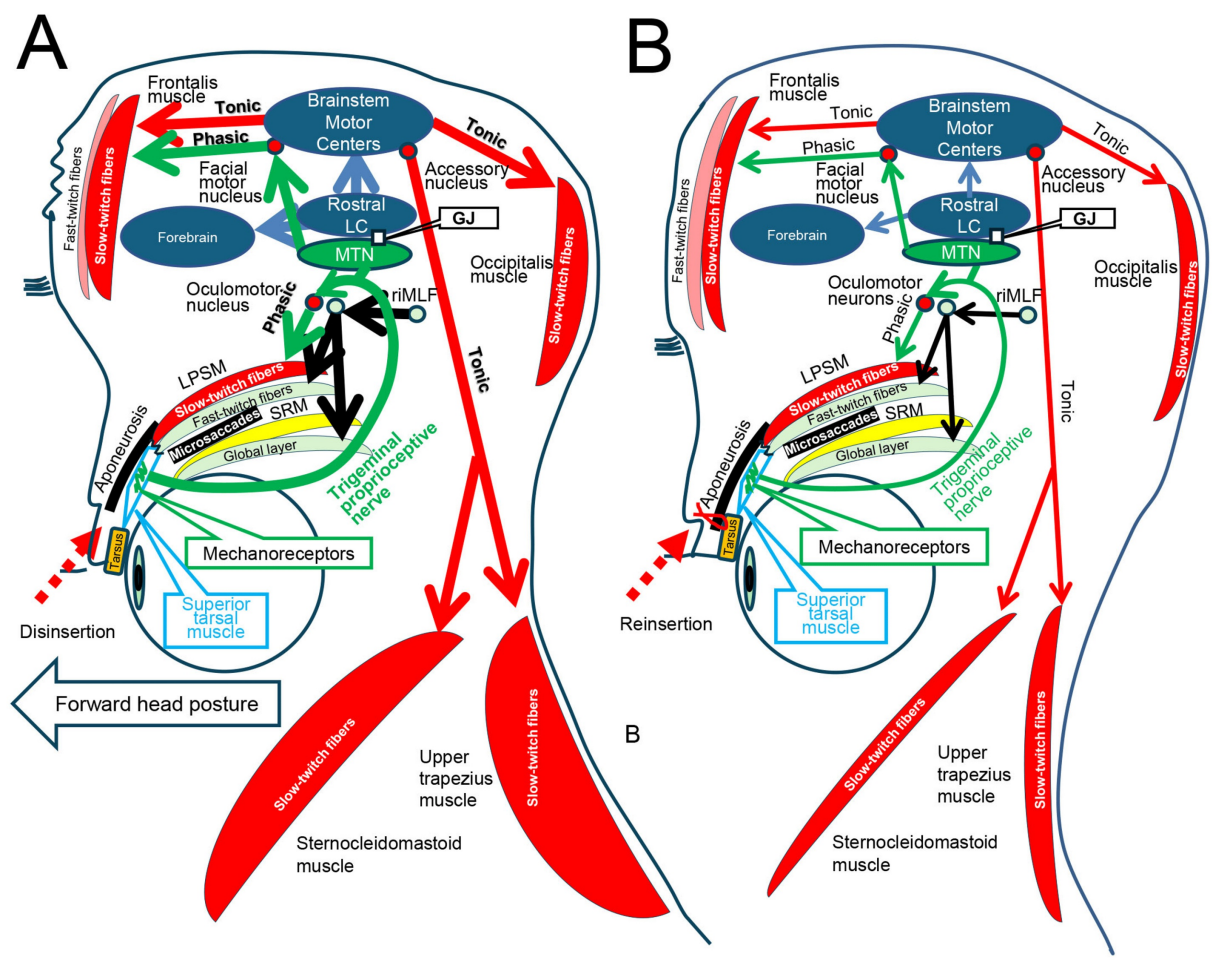
Diagram explaining the tonic reflex contractions of slow-twitch fibers in the upper trapezius and sternocleidomastoid muscles and the occipitofrontalis muscle. (A) Before surgery to reinsert the aponeurosis into the tarsus, the rostral interstitial nucleus of the medial longitudinal fasciculus (riMLF) and the oculomotor nucleus induce voluntary contractions and microsaccades of fast-twitch fibers in the levator palpebrae superioris muscle (LPSM) and the global layer of the superior rectus muscle (SRM) [1,2]. These voluntary contractions and microsaccades enhance the stretching of mechanoreceptors in the superior tarsal muscle [3, 4], increasing phasic reflex contractions (Phasic) of the slow-twitch fibers [5] in the LPSM [6] and frontalis muscle [7-9] via the mesencephalic trigeminal nucleus (MTN) and the oculomotor and facial motor nucleus [7]. They also increase tonic reflex contractions (Tonic) of slow-twitch fibers [5] in the occipitofrontalis [7-9] and the upper trapezius and sternocleidomastoid muscles via the MTN, rostral locus coeruleus (LC) [10], and brainstem motor centers [11], including the accessory nucleus. The MTN connects with the rostral locus coeruleus (LC) through gap junctions (GJ) [10] to also activate the forebrain for arousal [18]. The increased contractions of the upper trapezius and sternocleidomastoid muscles induce backward extension and protrusion of the head, resulting in a forward head posture. (B) After surgery to reinsert the aponeurosis to the tarsus [15,16]: Due to reduced stretching of mechanoreceptors in the superior tarsal muscle [3, 4], the phasic and tonic reflex contractions of slow-twitch fibers in the LPSM [6], occipitofrontalis muscle [7-9], and upper trapezius and sternocleidomastoid muscles are decreased. Image Credit: Kiyoshi Matsuo and Ai Kaneko

Figure 1 illustrates the neuroanatomy related to the phasic reflex contractions of slow-twitch fibers [5] in the LPSM [6] and the frontalis muscle [7-9]. It also depicts the tonic reflex contractions of slow-twitch fibers in the occipitofrontalis muscle [7-9], as well as in the upper trapezius and sternocleidomastoid muscles, both before and after surgery to reinsert the tarsus following the disinsertion of the aponeurosis.

This study was approved on February 2, 2016, by the Shinshu University School of Medicine Biological and Medical Research Ethics Committee (Permission number: 30869). Verbal or written informed consent was obtained from the patients for the publication of this case report and accompanying images.

Case 1

**Figure 2:**
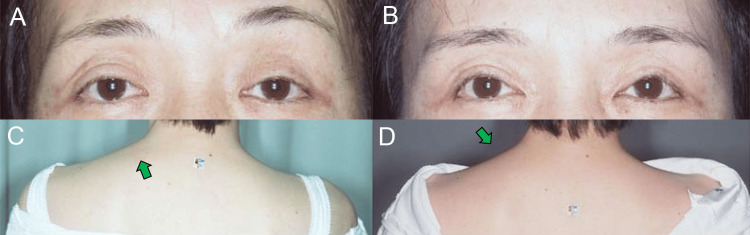
Case 1. (A, B) Preoperative primary gaze image shows changes in the position of the left eyebrow and upper trapezius muscle (indicated by a green arrow), transitioning from asymmetrically raised to even. (C, D) Postoperative photo reveals significant changes in the left eyebrow and upper trapezius muscle (indicated by a green arrow), shifting from contracted to relaxed occipitofrontalis and upper trapezius muscles.

A 48-year-old woman presented with asymmetrical aponeurosis disinsertion, predominantly affecting the left eyelid, and complained of left-sided tension-type headache (TTH) and neck pain. Her left eyebrow (Figure 2A) and upper trapezius muscle (Figure 2C) were more raised and contracted than the right side. After undergoing bilateral aponeurosis reinsertion surgery [15, 16], her left eyebrow (Figure 2B) and upper trapezius muscle (Figure 2D) were lowered and relaxed (Figure 1), which alleviated the left-sided TTH [9] and neck pain.

Case 2

**Figure 3:**
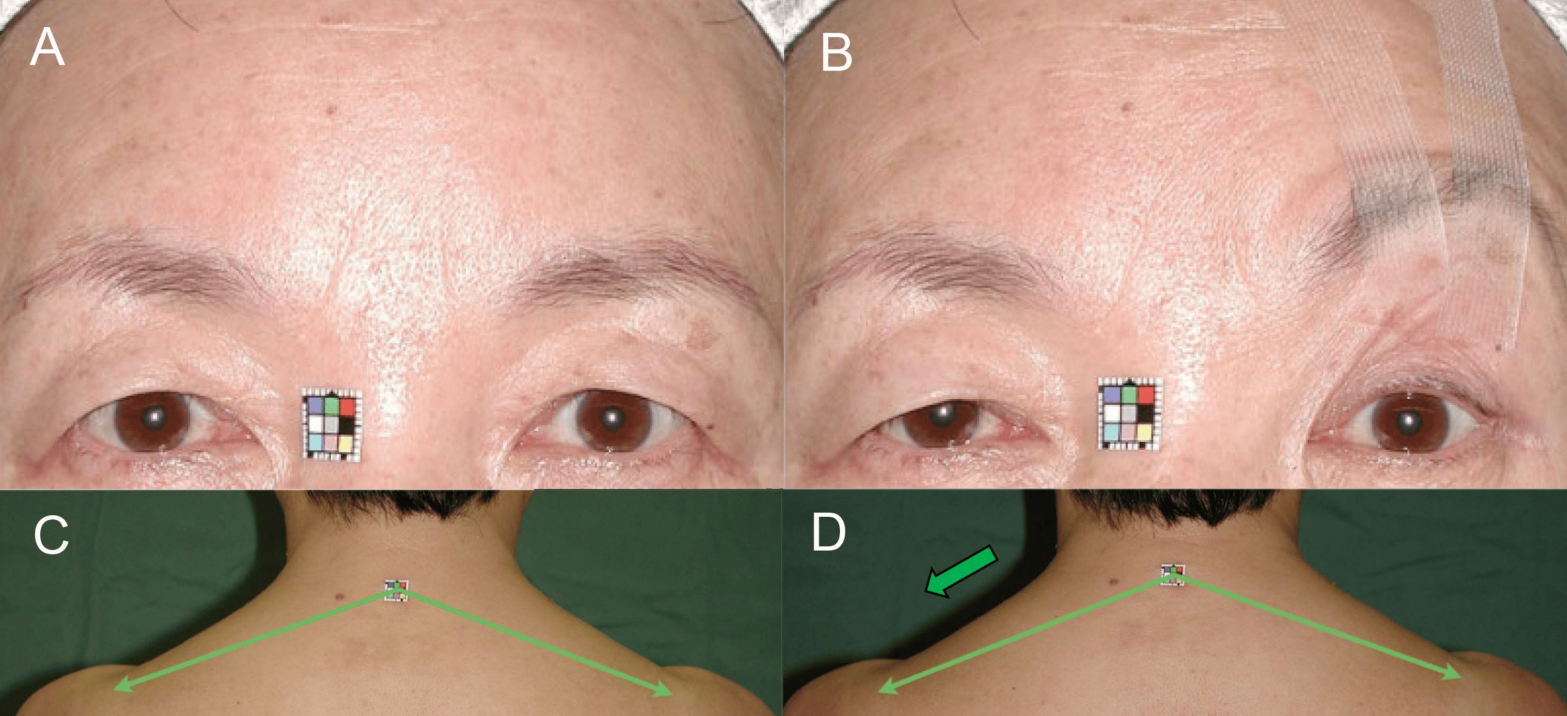
Case 2. (A, B) Primary gazing before and after unilateral eyebrow lifting with tape. Unilateral taping lowered the contralateral eyebrow and upper eyelid. (C, D) Before and after unilateral eyebrow lifting with tape. Thin, long green arrows indicate the distances between the seventh cervical spinous process and the acromion processes. Unilateral taping decreased the bilateral distances, with ipsilateral dominance indicated by the thick, short green arrow compared to a 1 cm square sizer attached to the skin on the seventh cervical spinous process.

A 55-year-old woman presented with bilateral aponeurosis disinsertion due to habitual eyelid stretching [9, 15]. Effortful upper eyelid retraction led to raised upper eyelids and eyebrows (Figure 3A) [9] and contracted bilateral upper trapezius muscles (Figure 3C), resulting in bilateral TTH [9] and neck pain. Unilateral eyebrow lifting with tape reduced mechanoreceptor stretching, decreasing tonic reflex contractions of slow-twitch fibers in the bilateral occipitofrontalis muscles (Figure 3B) [7-9] and upper trapezius muscles (Figure 3D), with a more pronounced effect on the taped side. To evaluate the decrease in tonic reflex contractions of the upper trapezius muscles, the bilateral distances between the seventh cervical spinous process and the acromion processes were measured photogrammetrically. Before the left eyebrow lifting with tape, the distances were 129 mm on the left and 128 mm on the right. After the taping in an upright sitting position, the distances increased to 138 mm on the left and 132 mm on the right.

Case 3

**Figure 4:**
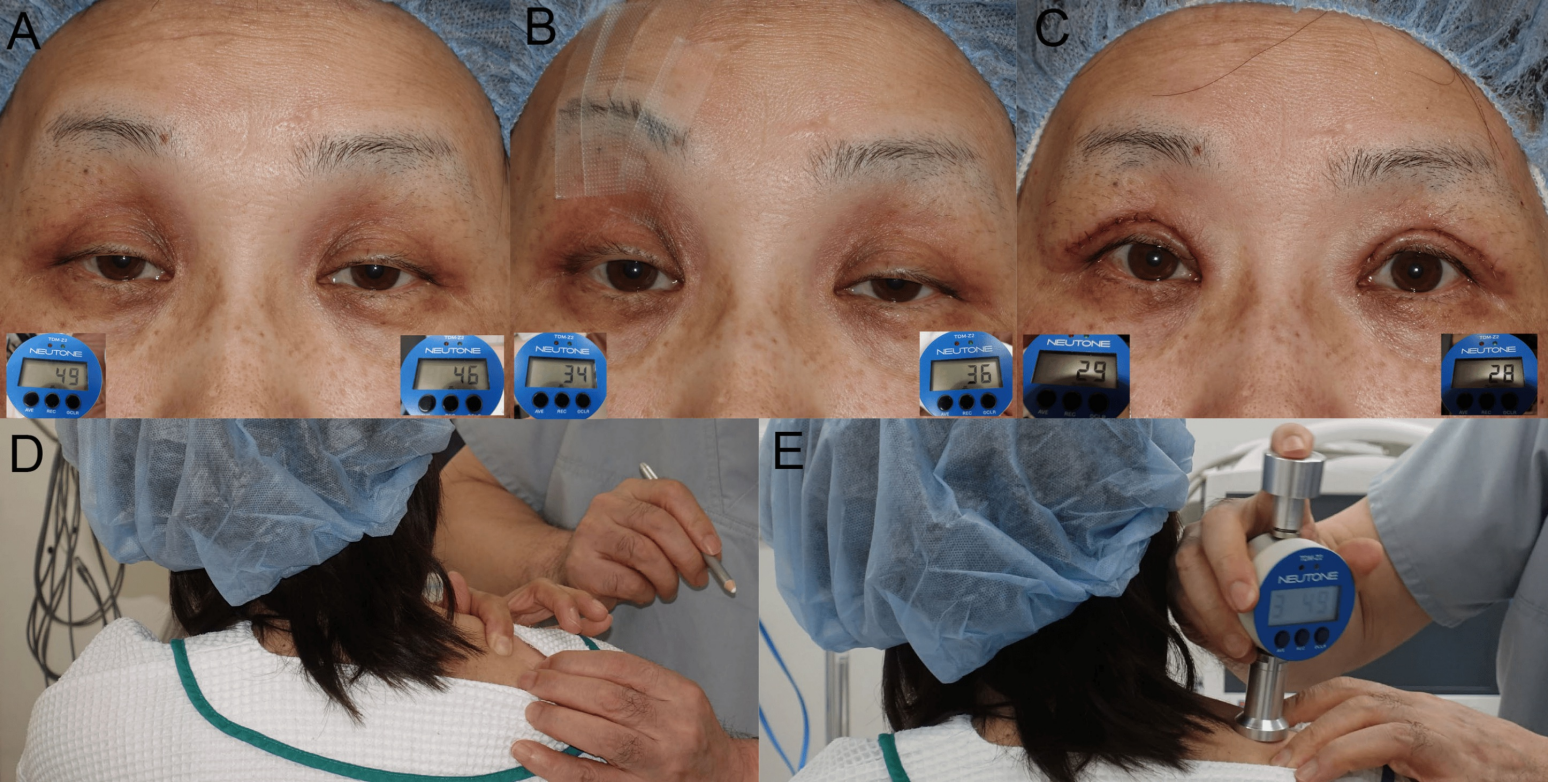
Case 3. (A) Primary gaze showing the scores of muscle hardness in the bilateral upper trapezius muscles. (B) Unilateral eyebrow lifting with tape and the scores of muscle hardness in the bilateral upper trapezius muscles. (C) Scores of muscle hardness in the bilateral upper trapezius muscles immediately after aponeurosis reinsertion surgery. (D) The hardest point in the upper trapezius muscles, as indicated by the patient. (E) Measurement of muscle hardness in the upper trapezius using the muscle hardness tester.

A 51-year-old woman presented with bilateral aponeurosis disinsertion and moderate elongation of the STM [9, 15, 16]. Effortful upper eyelid retraction did not open the upper eyelids over the pupils. It resulted in raised bilateral eyebrows (Figure 4A), contracting the bilateral occipitofrontalis and upper trapezius muscles and causing bilateral TTH [9] and neck pain. Unilateral eyebrow lifting with tape reduced mechanoreceptor stretching, decreasing tonic reflex contractions of slow-twitch fibers in the bilateral occipitofrontalis (Figure 4B) [9] and upper trapezius muscles, with a more pronounced effect on the taped side. Bilateral aponeurosis reinsertion surgery further reduced mechanoreceptor stretching, decreasing tonic reflex contractions of slow-twitch fibers [5] in the bilateral occipitofrontalis (Figure 4C) [7-9] and upper trapezius muscles (Figure 1), effectively curing the TTH [9] and neck pain. The contractions of the upper trapezius muscles were measured using a muscle hardness tester (TDM-N1, Sato Shouji Inc., Kawasaki, Japan) [17]. While the score of muscle hardness was not quantitative but relative, the hardest point in the upper trapezius muscles, near the acromion process, was identified by the patient (Figure 4D) and measured (Figure 4E) bilaterally during primary gazing (Right: 48, Left: 46) (Figure 4A), after unilateral eyebrow lifting with tape (Right: 34, Left: 36) (Figure 4B), and immediately after surgery (Right: 29, Left: 28) (Figure 4C). The unilateral eyebrow lifting with tape decreased muscle hardness bilaterally with ipsilateral dominance, and the surgery further decreased muscle hardness bilaterally.

Case 4

**Figure 5:**
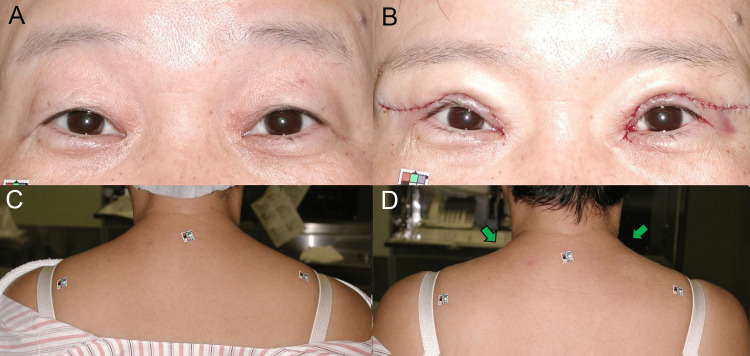
Case 4. (A) Primary gaze showing tonically elevated eyebrows. (B) Immediately after surgery for debulking to reduce the volume and weight of the upper eyelids and reinserting the aponeuroses to the tarsi. (C) Primary gaze showing the straight outline of bilaterally contracted upper trapezius muscles. (D) Immediately after surgery, the straight outlines of the upper trapezius muscles changed to concave (indicated by green arrows) due to relaxation.

A 60-year-old woman presented with tonically raised bilateral eyebrows due to reflexively contracted occipitofrontalis muscles (Figure 5A) [7-9] and straight outlines of the upper trapezius muscles from their tonic contraction (Figure 5C). Immediately after bilateral debulking and aponeurosis reinsertion surgery, the eyebrows were lowered (Figure 5B), and the straight outlines of the upper trapezius muscles became concave (Figure 5D), alleviating the tension-type headaches (TTH) [9] and neck pain.

Case 5

**Figure 6:**
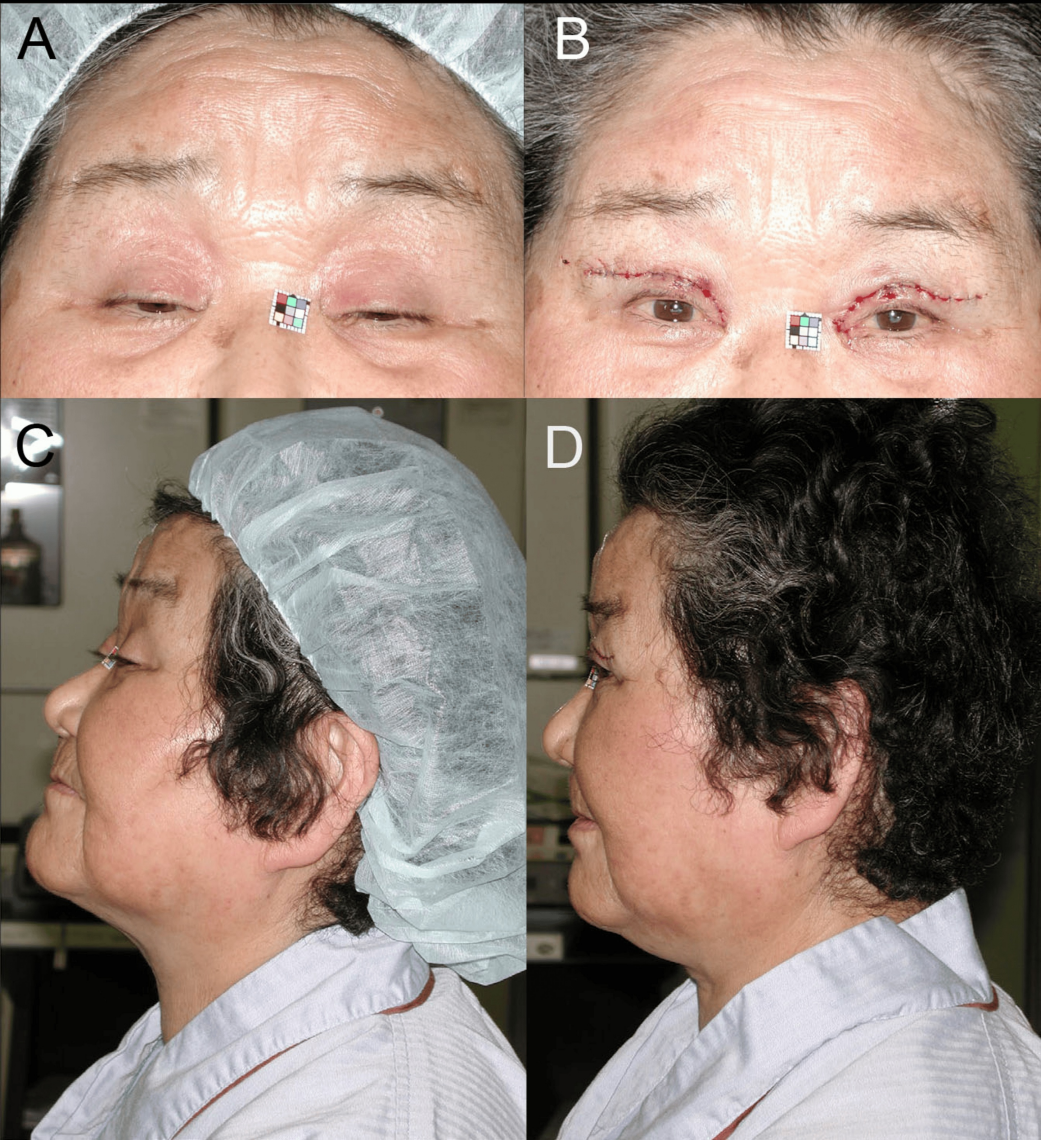
Case 5. (A) Primary gaze with maximally lifted eyebrows before surgery. (B) Primary gaze immediately after surgery for reinserting the aponeuroses to the tarsi. (C) Left lateral view in primary gaze before surgery, showing the head extended backward and protruded. (D) Left lateral view in primary gaze immediately after surgery, showing that the extension and protrusion of her head were well corrected. Note that the raised hairline was lowered through surgery.

An 85-year-old woman presented with aponeuroses disinsertion and elongated STMs [15, 16]. Her bilateral eyebrows were tonically lifted to their maximum (Figure 6A), and her head was extended backward and protruded due to increased tonic reflex contractions of the upper trapezius and sternocleidomastoid muscles (Figure 1A and Figure 6C). Immediately after bilateral aponeurosis reinsertion surgery, her eyebrows were lowered due to relaxation of the occipitofrontalis muscle (Figure 6B) [9], and the extension and protrusion of her head were reduced due to relaxation of the upper trapezius and sternocleidomastoid muscles (Figure 6D), relieving the tension-type headaches (TTH) and neck pain (Figure 1B).

## Discussion

Unilaterally increased stretching of the mechanoreceptors in the STM [3, 4] due to increased voluntary contractions and microsaccades of fast-twitch fibers in the LPSM and GLSRM [1, 2] in patients with asymmetrical aponeurosis disinsertion appeared to ipsilaterally increase the tonic reflex contractions of slow-twitch fibers in the occipitofrontalis muscle [5, 7-9] and upper trapezius muscle (Figure 1A and Figure 2A, 2C). This process occurs as part of the eye-eyelid-eyebrow-head coordinated movements via the mesencephalic trigeminal nucleus (MTN), rostral locus coeruleus [10], and brainstem motor centers [11], including the facial and accessory motor nuclei, leading to ipsilateral tension-type headache (TTH) [9] and neck pain [19, 20]. Conversely, unilaterally decreased stretching of the mechanoreceptors in the STM, achieved through lifting the eyebrow with tape (Figure 3B, 3D and Figure 4B), and bilaterally decreased stretching of the mechanoreceptors in the STMs, achieved through surgical reinsertion of the bilateral aponeuroses to the tarsi (Figure 1B, Figure 4C, Figure 5B, 5D) [9], resulted in decreased tonic reflex contractions of slow-twitch fibers in the occipitofrontalis muscle and upper trapezius muscle, relieving both ipsilateral and bilateral TTH [9] and neck pain. Thus, the five cases suggest that neck pain may simply be due to increased tonic reflex contractions of slow-twitch fibers in the upper trapezius muscle, driven by increased stretching of mechanoreceptors in the STM in patients with aponeurosis disinsertion via the MTN, rostral locus coeruleus, and brainstem motor centers (Figure 1A).

The reason for the change in head posture was not simply due to the correction of blepharoptosis (Figure 6). The lowering of the raised hairline through surgery led to the presence of increased stretching of mechanoreceptors in the superior tarsal muscle [3, 4, 9, 10] to the extent of more than a 60° upgaze. This stretching induces reflex contractions of slow-twitch fibers in the occipitalis muscle to raise the hairline upward [8, 9] and appears to trigger reflex contractions of the upper trapezius and sternocleidomastoid muscles as well, resulting in forward head posture (Figure 1). In patients with severe blepharoptosis, whenever they look upward and outward, their eyes do not move, but their heads do. This is likely due to the maximally increased contractions of fast-twitch fibers in the LPSM and the global layer of the superior rectus muscle, which may restrict eye movement by involving the global layers of other extraocular muscles in primary gaze. The LPSM, originating from the sphenoid bone and inserting into the upper eyelid tarsi as levator aponeurosis (Figure 1), is oriented at approximately 23 degrees laterally. Thus, lateral gaze induces asymmetrical stretching of mechanoreceptors in the STMs. The mechanoreceptors in the STM on the side of lateral gaze are stretched more, which may increase not only reflex contractions of slow-twitch fibers in the LPSM and occipitofrontalis muscle to elevate the eyelid, eyebrow, and scalp [9], but also reflex contraction of the contralateral sternocleidomastoid muscle to move the head laterally. Bilaterally increased mechanoreceptor stretching may enhance bilateral contractions of the upper trapezius muscles to extend the head backward and the sternocleidomastoid muscles to protrude the head, likely playing a crucial role in forward head posture.

We previously reported that preoperatively, to confirm whether the increased stretching of the mechanoreceptors in the STM in patients with aponeurosis disinsertion [15, 16] induced tonic reflex contractions of slow-twitch fibers in the occipitofrontalis muscle [7-9], leading to TTH, administration of 1% phenylephrine to the upper fornix to contract the STM is a versatile procedure. Contraction of the smooth muscle fibers in the STM desensitizes the intramuscular trigeminal proprioceptive fibers, allowing the pharmacologically contracted STM to transmit the retractile power of the LPSM instead of the aponeurosis. Upper eyelid opening with the contracted STM reduces the stretching of the mechanoreceptors in the STM [3, 4], thereby decreasing tonic reflex contractions of slow-twitch fibers in the occipitofrontalis muscle and relieving TTH [9]. Thus, in addition to lifting the eyebrow with tape, we could confirm the etiology of neck pain by administering 1% phenylephrine to the upper fornix to contract the STM.

The locus coeruleus sends projections to the forebrain, brainstem, cerebellum, and spinal cord to regulate arousal, sympathetic tone, postural muscle tone, and pain modulation (Figure 1A, 1B) [10, 11, 18]. The five cases indicated that the rostral locus coeruleus [10], activated by stretching of mechanoreceptors in the STM due to phasic voluntary contractions and microsaccades of the fast-twitch fibers in the LPSM and GLSRM [1, 2], appeared to send projections to the brainstem motor centers [11], including the motor nuclei, such as the accessory nucleus, to induce tonic reflex contractions of slow-twitch fibers in the upper trapezius and sternocleidomastoid muscles, as well as the facial motor nucleus [12], to induce tonic reflex contractions of slow-twitch fibers in the occipitofrontalis muscle [7-9], orbital orbicularis oculi [7, 13, 14], and facial expression muscles [12], facilitating coordinated movements of the eye, eyelid, eyebrow, face, and head.

Stretching of mechanoreceptors in the STM regulates physiological arousal, leading to increased forebrain blood flow and sympathetic activation, such as palmar sweating [18], which in turn increases microsaccade velocity (Figure 1A, 1B) [1]. Neck pain is the fourth leading cause of disability, often work-related, and is commonly caused by prolonged static positions and repetitive tasks [19, 20]. Increased mechanoreceptor stretching due to the increased voluntary contractions of fast-twitch fibers in the LPSM and GLSRM and microsaccade velocity [1, 2] during primary gazing at visual display terminals may not only activate forebrain blood flow [18] but also reflexively contract slow-twitch fibers in the upper trapezius muscle to prevent the head from drooping forward, potentially resulting in neck pain (Figure 1A, 1B).

If we may express our thoughts candidly, we observed coordinated movements of the eyes, eyelids, eyebrows, and head in a 26-day-old boy (Video 1). Babies typically begin lifting their heads at approximately one month of age. Careful observation of this developmental milestone reveals that during head lifting, they consistently gaze upward while raising their eyebrows. When the upward movement of the eyes, increased eyelid retraction, and raised eyebrows are interrupted, their heads abruptly drop, demonstrating the coordination between the eyes, eyelids, eyebrows, and head.

**Video 1:** A 26-day-old boy is undergoing neck-sitting training. Upgaze enhances the stretching of mechanoreceptors in the STM [3, 4], inducing reflex contractions of the LPSM [6], occipitofrontalis muscle [7-9], and upper trapezius muscle as part of the coordinated movements of the eyes, eyelids, eyebrows, and head. In contrast, downgaze abruptly eliminates all reflex contractions. Written informed consent to include this video in the published article was obtained from the patient's legal guardian.

## Conclusions

Individuals who habitually stretch their eyelids, such as when applying contact lenses or eyedrops, often experience disinsertion of the aponeuroses from the tarsi, leading to increased mechanoreceptor stretching. The neck pain and TTH resulting from increased tonic reflex contractions of slow-twitch fibers in the upper trapezius muscle and occipitofrontalis muscle in individuals with aponeurosis disinsertion may be treatable following preoperative diagnostic confirmations and aponeurosis fixation surgery.
